# Linking Newborns and Mothers to Enable the Study of Inter-generational Health Outcomes: Evidence from Nationwide Medicaid Data

**DOI:** 10.21203/rs.3.rs-5327524/v1

**Published:** 2024-11-14

**Authors:** Lilla Orr, Basil Seif, Sun Jeon, Elisa Cascardi, Sakshina Bhatt, Jonas Swartz, Maria Isabel Rodriguez, Lee Sanders, Fernando Mendoza, Jens Hainmueller

**Affiliations:** ∗ University of Richmond; † Stanford University; ‡ Duke University; § Oregon Health and Science University

## Abstract

Linking mothers to their newborns in health records is crucial for understanding the impact of policies, programs, and medical treatments on inter-generational health outcomes. While previous studies have used shared identifiers like names or addresses for linkage, such data are often unavailable in Medicaid records due to privacy concerns. We present a scalable framework and linking algorithm using Medicaid MAX and TAF claims data—lacking direct identifiers—that connects mothers and infants while ensuring privacy protection. Our method accommodates variations in Medicaid records over time and across states, supporting matches at different levels of stringency. Using data from all 50 states over 19 years, our algorithm linked 11.68 million mother-infant dyads, covering 68% of Medicaid-enrolled infants, over 30% of all U.S. infants. We provide our code to facilitate research on social determinants of health and the inter-generational effects of U.S. public policy.

## Introduction

Understanding the health of infants and young children requires considering family contexts. However, to protect privacy, health records often lack linkages or shared identifiers, such as names or addresses, between mothers and newborns, making it challenging to study family and intergenerational health effects. For births covered by Medicaid, we introduce a method to link infants and mothers^1^ without relying on direct identifiers. This is significant because Medicaid has covered approximately 42% of U.S. births in recent decades (20; 15). Accurately establishing these connections while maintaining privacy is crucial for research on social determinants of early childhood health (9; 13), the impact of medication use during pregnancy on child health (14; 25), and the intergenerational effects of public policy (24; 19). Given that Medicaid funds nearly half of all births nationwide, analyzing Medicaid beneficiaries provides valuable insights into the health of mothers and their children. Although this population is not representative of the entire nation, it is critical for evaluating public policies aimed at reducing health disparities and addressing potential unintended consequences for low-income Americans.

Our linking approach expands on previous work in several ways. First, building on earlier research (4; 23; 2; 12; 16; 17), we develop a detailed framework for identifying newborns and deliveries that accounts for variations in Medicaid records over time and across states. This framework utilizes an extensive set of diagnosis and procedure codes as well as enrollment records. It is particularly important for newborns because, in several states, their care is billed under the mother’s record for a period after birth, meaning they cannot be identified using diagnosis and procedure codes appearing in their own Medicaid claims. Addressing these discrepancies is essential for applying our algorithm nationwide and over time, covering the transition from the Medicaid Analytic Extract (MAX) to the Transformed Medicaid Statistical Information System (TAF) Analytic Files (21; 16; 10). This comprehensive approach enables large-scale analyses of public policies that impact low-income families across different domains, such as housing, education, and health.

Second, we validate our procedures for identifying newborns and deliveries in Medicaid claims by comparing the number of identified newborns and deliveries to expected counts based on micro-data from the National Vital Statistics System (NVSS).

Third, we develop and implement a deterministic linking algorithm that utilizes dates, ZIP codes, and, when available, race, ethnicity, and case IDs, sometimes assigned at the household level. A key advantage of our algorithm is that it does not require Medicaid claims to be linked to vital records or other beneficiary-level data, as is often needed for identifying family units within Medicaid (22; 2; 17). It also does not rely on electronic health records, private insurance data, or other non-governmental sources (6; 18; 7; 26), thereby protecting the privacy of beneficiaries. Additionally, it allows researchers to establish matches at varying levels of stringency.

Fourth, our approach is designed to include groups often excluded from infant-mother linkages, such as those without full Medicaid coverage^2^ or those enrolled for only short periods (21). Excluding these groups can limit the scope of analyses focused on social determinants of health. We include these groups and confirm that our procedure yields high identification rates across diverse demographic groups. Our algorithm is designed to be as inclusive as possible, while also being adaptable for researchers with different needs.^3^ Our publicly available code is built with flexibility in mind.

From 2011 to 2019, across all fifty states, we identified the Medicaid beneficiaries who gave birth to 68% of the infants enrolled in Medicaid, linking 11.68 million infant-mother dyads. Our linked cohort also represents the broader population of Medicaid beneficiaries on key observable characteristics including race and ethnicity, age, gender, and region.

## Data and Methods

### 1 Data

Our analysis draws on data from Medicaid Analytic Extract (MAX) from 2011 to 2014 and Transformed Medicaid Statistical Information System (T-MSIS) Analytic Files (TAF) spanning from 2014 to 2019. Our MAX data analysis utilizes Personal Summary, Inpatient, and Other Services files. Similarly, our TAF data analysis utilize Demographic Eligibility, Inpatient and Other Services files. All data were acquired directly from Centers for Medicaid and Medicare Services (CMS). Details regarding data acquisition can be found in Appendix 1.

We do not rely no any external information regarding beneficiaries. We use information from providers to determine facility ZIP codes. For physicians and physician groups providing care for deliveries and newborns, we link the National Provider Identifier (NPI) appearing in MAX and TAF claims to records from the National Plan and Provider Enumeration System (NPPES). Specifically, we utilize the ZIP code of the employment address that was registered for each NPI during the year in which the claim was filed. For further details on our processing of NPPES data, see Data Acquisition Appendix 1.

Our methodology for linking mothers and infants involves three steps. First, we identify deliveries of living singleton newborns. Second, we identify singleton newborns. Third, we link the two groups using our linking algorithm.

### 2 Identification of Deliveries

To identify deliveries, we leverage a comprehensive list of International Classification of Diseases (ICD-9 and ICD-10) and Current Procedural Terminology (CPT) codes to capture claims indicating deliveries resulting in live births. Our list includes 96 distinct codes, detailed in Appendix 2, which are derived from Auty et al. (4, 3, 5) method 4 and Sarayani et al. (23). To avoid false positives, we excluded codes that may suggest delivery but are also commonly used in claims for antepartum or postpartum care. Further information on our process for refining this code list is available in Appendix 2.

Using this code list, we identified all claims^4^ that list these codes across all inpatient and other services files. We then identified the beneficiary of each claim and retained those who were between the ages of 12 and 50 at the time of the claim.

Next, we mapped the delivery claims for each beneficiary to unique, distinct deliveries, as a single delivery may generate multiple claims on different days, and some beneficiaries may have had multiple deliveries over our nine-year study period. We used a hierarchical clustering algorithm to group the delivery claims into unique delivery clusters. Clustering was based on dates, with all claims within a given time period assumed to be associated with the same delivery. We required that the midpoint of each delivery period be at least 270 days apart from the midpoint of any other delivery period to ensure that data errors would not lead us to incorrectly assume that a beneficiary had two deliveries resulting in live births within a nine-month span.

Finally, we validated our procedure by comparing the number of deliveries we identified (i.e., the number of delivery claim clusters) to the number of deliveries recorded in the CDC’s National Vital Statistics System (NVSS), for which Medicaid is listed as a payer. We conducted this comparison at the state-year level to account for variations in Medicaid claims reporting, diagnostic and procedure code usage, and data processing systems at the state and federal levels over time and space.

For each identified delivery, we estimated the time and place of the delivery using the delivery claims, as well as the residential ZIP code, case ID number, and racial or ethnic identity of the mother based on enrollment data. We used service dates to estimate the time windows during which deliveries took place and NPIs to estimate facility ZIP codes. We removed invalid ZIP codes and case ID numbers assigned to more than 10 beneficiaries in a given state-year, as we do not expect these to have been assigned at the household level. We also excluded indicators of multi-racial identity, as we anticipate relatively low congruence between the racial and ethnic identity of infants and mothers (as recorded in Medicaid enrollment data) when either is reported as multi-racial. Data processing details are outlined in Appendix 4.

### 3 Identification of Newborns

To identify newborns, we utilized ICD-9, ICD-10, and CPT codes that indicate care specific to newborns. We compiled these codes by integrating lists from various sources, including (8), ResDAC resources for identifying newborn care^5^, and an original review of annual medical coding manuals. The complete list of codes is provided in Appendix 3. We identified all claims containing these codes or an indicator of inpatient admissions of newborn infant, billed for beneficiaries from their birth date up to 7 days post-birth.

However, this method alone is insufficient to identify newborns nationwide. In several states, care for newborns is often billed under their mother’s coverage, making it impossible to directly observe the identity of the newborn receiving care based solely on claims data (CMS).

To address this limitation, we employed an additional method that does not rely on diagnosis and procedure codes. Specifically, we identified state-years where the number of newborns detected using our list of diagnosis and procedure codes was less than 95% of the total number of newborns recorded in the NVSS records. For these state-years (listed in Appendix 3), we included all beneficiaries who were enrolled in Medicaid before or up to one day after their date of birth in our universe of newborns, utilizing enrollment dates from the enrollment files. The rationale is that newborns enrolled in Medicaid at or near the time of birth should be considered as candidates for matching because they are likely born to mothers who are also included in the Medicaid claims.

After establishing the comprehensive list of newborns, we identified data elements essential for linking them to their mothers. We used enrollment records to capture their date of birth, residential ZIP code, and racial and ethnic identity. We excluded invalid ZIP codes and beneficiary IDs assigned to more than 10 individuals and flagged indicators of multi-racial identity. For cases with claims indicating care at the time of birth, we estimated the facility ZIP code using the National Provider Identifiers (NPIs) on these claims to infer the location of birth.

### 4 Matching Algorithm

To link mothers to newborns, we use a greedy deterministic matching algorithm, where each unmatched infant can be linked to only one unmatched delivery and vice versa. The matching is based on the state of residence, newborn’s date of birth, delivery date window, and four demographic variables: (1) residential ZIP code of the beneficiary (mother or newborn), (2) ZIP code of the facility where the delivery or newborn care occurred, (3) race/ethnicity of the beneficiary, and (4) beneficiary’s case number. The underlying premise is that infants must be born at approximately the same time and place as the delivery occurs, and the beneficiaries should share attributes that we expect mothers and infants to have in common (such as ZIP codes).

We exclude newborns for which we cannot confidently determine the birth date and deliveries for which we cannot identify a narrow range of plausible dates for the delivery window. For the remaining matching variables, we allow for missing data. A summary of missing data is available in Appendix 4.

The matching algorithm proceeds in six phases, each involving progressively less restrictive criteria. Across all phases, we always require that the state of the newborn matches the state of the mother and that the newborn’s date of birth falls within a narrow range around the delivery date window. Matches are accepted only if there is a unique mother-newborn pair that satisfies the criteria. This helps prevent incorrect matches. Any linked newborns and deliveries are removed from the unmatched pool, and the algorithm proceeds to identify unique matches using slightly less stringent criteria. A detailed summary of the criteria for each matching phase is provided in Appendix 5.

In phase one, we impose the most stringent criteria. We require that the newborn’s date of birth falls within the estimated delivery date range and that the newborn and mother share the same unique case number. A shared case ID is a strong indicator of a shared household^6^. Additionally, we require that they share the same facility ZIP code, residential ZIP code, and racial or ethnic identity. For these three variables, we iterate through eight steps, requiring that newborns and mothers match on all three, any two, any one, or none of these variables (see Appendix 5 for details).

In phases two and three, we repeat the steps from phase one but allow for a one- or two-day deviation from the estimated delivery date range. In phases four through six, we relax the criteria further by allowing for missing or conflicting case IDs. This is necessary because some states do not use case ID numbers, and others may assign different numbers to parents and their children. We begin by looking for unique matches for infants born within the estimated delivery date range, with shared residential and facility ZIP codes and race or ethnicity (phase four). Then, the allowable date range is expanded by one day (phase five) and two days (phase six), as in the first three phases.

The sequential, deterministic nature of this algorithm ensures high transparency. Researchers can exclude dyads from specific matching phases from their analyses if they believe the matches were made using insufficiently stringent criteria.

## Results

### 1 Identification: Deliveries and Newborns

**Figure 1:**
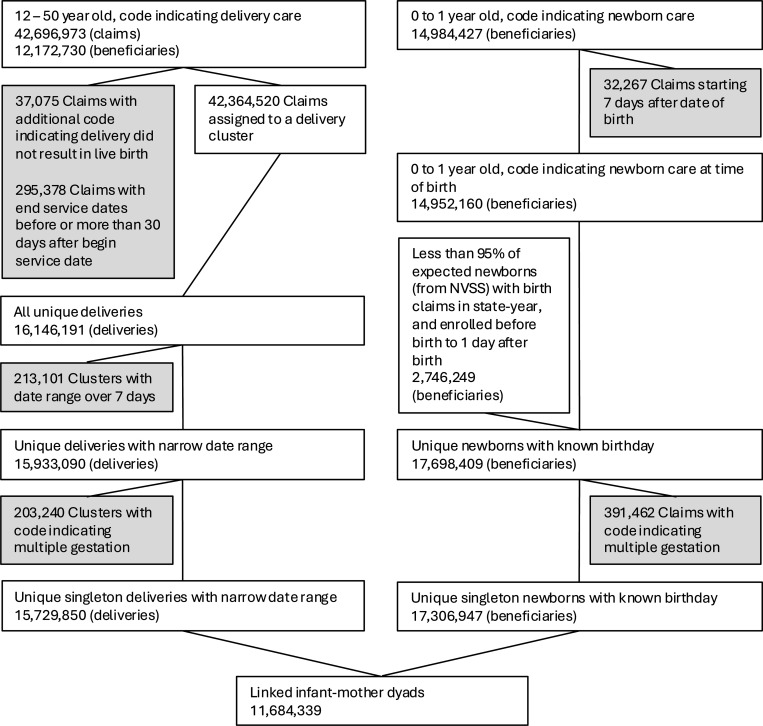
Total Number of Deliveries and Newborns Included in the Analysis Note: Total sample size at each stage of data processing. Shaded boxes indicate exclusions from further analysis.

The aggregated results from our identification of deliveries and newborns, as well as the linking process between mothers and newborns, are presented in Figure 1.

Across nine years of nationwide Medicaid records, our delivery identification method yields 12,172,730 beneficiaries aged 12 to 50 years with claims indicating they received care for a delivery resulting in a live birth. After consolidating these delivery-related claims into unique deliveries using our hierarchical clustering algorithm, we identify a total of 16,146,191 unique deliveries. A detailed description of the delivery claim clusters can be found in Appendix 2. For the subsequent matching, we exclude 213,101 deliveries (1.32%) for which we are unable to estimate a delivery time window of 7 or fewer days, and 203,240 deliveries (1.26%) that appear to have resulted in multiple gestation, based on diagnosis and procedure codes listed in Appendix 3, as our focus is on singleton births.

Using the same universe of Medicaid claims, our newborn identification method identifies 17,698,409 beneficiaries who were covered by Medicaid at the time of birth. This includes 14,952,160 newborns with a birth-related claim under their own beneficiary ID, as well as 2,746,249 beneficiaries from states and years where care for newborns was likely not billed directly to them. In these cases, we identified the newborns based on their Medicaid enrollment at the time of birth. For the subsequent matching, we additionally exclude 391,462 newborns (2.21%) who do not appear to be singletons, based on diagnosis and procedure codes listed in Appendix 3.

While the number of newborns roughly matches the number of deliveries, there are slightly more newborns identified. This discrepancy is likely due to cases where some newborns are Medicaid beneficiaries at birth, but their mothers did not use Medicaid insurance for their deliveries. These newborns will later be excluded during the matching process because they cannot be linked to a corresponding delivery.

**Figure 2:**
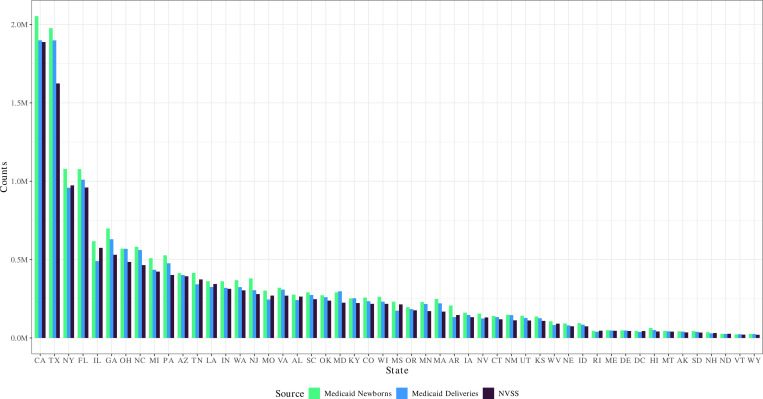
Total Number of Deliveries and Newborns by State Note: Total number of deliveries and newborns identified prior to removing multiple gestation births, compared to the expected number of deliveries (NVSS). States are listed in order based on the expected number of deliveries.

Next, we compare the number of identified deliveries and newborns against the number of deliveries and newborns covered by Medicaid as reported in the NVSS data. Figure 2 shows this comparison at the state level. Note that this comparison is based on the numbers before excluding multiple gestation newborns and deliveries, as these cannot be separately identified in the NVSS data.

We find a high degree of congruence between our identification of newborns and deliveries in the Medicaid data and the numbers reported in the NVSS across all states. These patterns are consistent across states and over time, as discussed in Appendices 2 and 3. Our identification consistently yields slightly higher numbers of deliveries than those reported in the NVSS. Overall, our identification using Medicaid claims yields 9.28% more deliveries than reported in the NVSS data as being covered by Medicaid. For newborns, our identification yields 19.78% more newborns than reported in the NVSS. This over-coverage is likely due to newborns enrolled in Medicaid at birth whose mothers did not use Medicaid for the delivery, misreporting in the payer variable in the NVSS data, or other discrepancies between the two datasets.^7^ However, this over-coverage is not a significant problem for matching, as newborns not born under Medicaid will go unmatched.

### 2 Linked Cohort

**Figure 3:**
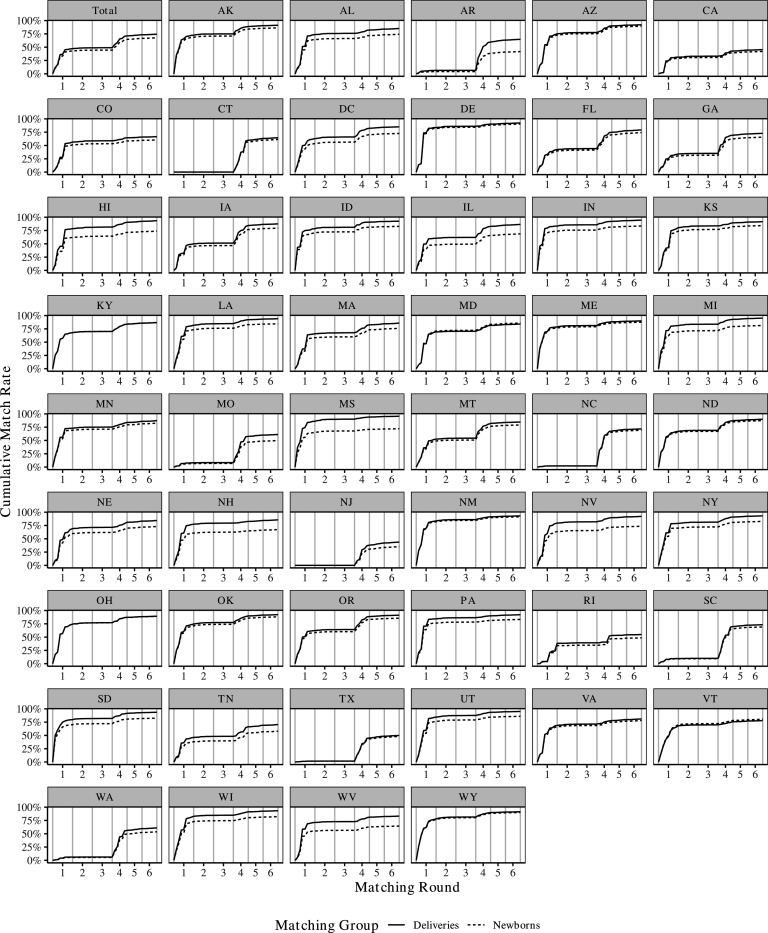
Cumulative Match Rate Nationwide and by State Note: Each plot shows the cumulative percentage of deliveries (solid lines) and newborns (dotted lines) linked at each step of the matching algorithm. Divergences between the lines indicate instances where there were more newborns than deliveries eligible for matching. The first plot shows nationwide results, while the remaining plots show state-level results.

Figure 3 displays the results of the matching algorithm by phase, both for the total cohort (top-left panel) and for each state. Detailed results for every step are provided in Appendix 5. Overall, through all six phases, our matching algorithm results in a total of 11,684,339 mother-newborn dyads. This represents 74.28% of deliveries matched to newborns and 67.51% of newborns matched to a delivery.^8^

Encouragingly, a substantial portion of these matches are based on very strict criteria. In phase one, which requires newborns and mothers to match on delivery time, case numbers, and combinations of residential ZIP code, facility ZIP code, and race/ethnicity, we identify 7,432,402 mother-newborn dyads. This corresponds to 42.94% of newborns and 47.25% of deliveries.

Repeating the steps from phase one but expanding the delivery window by 1 and then 2 days in phases two and three results in only a limited number of additional matches. At the end of phase two, we have identified 7,661,860 mother-newborn dyads, and at the end of phase three, 7,689,211 dyads. Since all these matches are based on exact case number matches, there is reason to be confident in their accuracy.

What is missing from phases one to three are states that do not assign case ID numbers at the household level, including states such as Texas, New Jersey, and Connecticut. To capture matches in these states, phases four to six exclude the case ID requirement, instead matching mothers and newborns based on date, location, and race/ethnicity. As shown in Figure 3, this adjustment results in a significant number of additional matches. By the end of phase four, there are 11,190,695 linked mother-newborn dyads, representing 71.14% of all deliveries and 64.66% percent of all newborns. This increase is most pronounced in states that do not report case ID numbers in the Medicaid data. At the end of phases five and six, there are 11,525,870 and 11,684,339 mother-newborn dyads, respectively.

Figure 3 illustrates that the algorithm is flexible enough to identify a large number of family units in every state, despite variations in data quality and reporting procedures. For newborns, linking rates range from 35.08% in New Jersey to 91.42% in New Mexico, with a median rate of 79.09%. State-specific linking rates at the end of phases three and six are available in Appendix 5.

**Figure 4:**
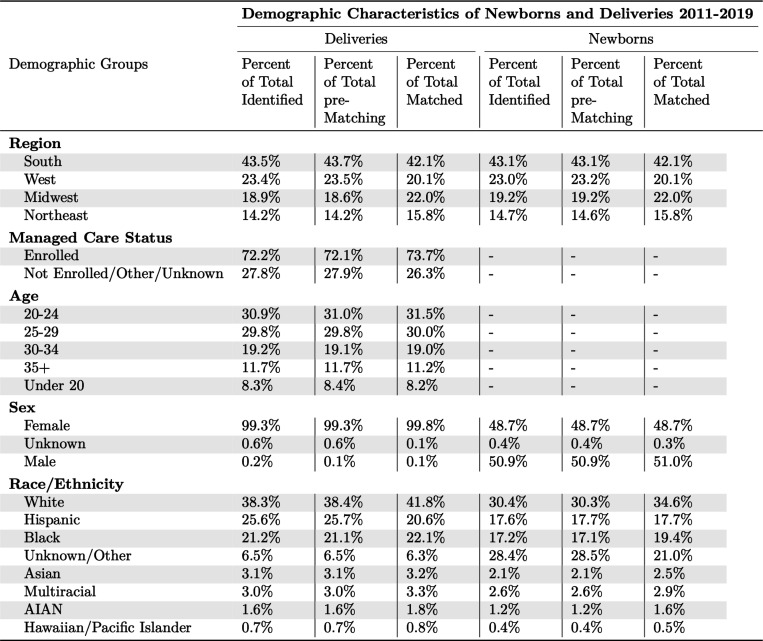
Demographic Differences Between Identified and Matched Beneficiaries Note: Age and rates of enrollment in managed care plans are not summarized for newborns.

To assess the representativeness of the linked cohort compared to the total universe of deliveries and newborns, we examined whether the linked cohort broadly represents all Medicaid beneficiaries on observable dimensions. Figure 4 presents the demographics of these beneficiaries before and after linking. For both deliveries and newborns, there is no meaningful difference between the demographics of those we identified and those we attempted to match. The linked cohort is slightly more likely to include beneficiaries from Midwestern and North Eastern states and to be White or Black, and slightly less likely to include those from Southern and Western states or those who are Hispanic or have unknown race/ethnicity.

## Discussion

Our method of identifying infant-mother dyads in nationwide Medicaid data offers significant advancements over standard practices and serves as a guide for researchers aiming to incorporate birth circumstances into the study of children’s health or explore the intergenerational health impacts of public policy. This method enables large-scale, nationwide, longitudinal analysis while protecting the privacy of Medicaid beneficiaries. By pooling data across time and geographic regions, it also facilitates research on small, often underrepresented groups that are typically excluded from policy analysis due to sample size limitations.

These advancements are made possible by not relying on vital records (11), harmonizing MAX and TAF data, and accounting for substantial variations in how states enroll newborns in Medicaid and record household units. Nationwide, longitudinal analysis of family units using Medicaid claims data is both feasible and essential for advancing our understanding of maternal and child health. We hope that our publicly available code will empower researchers to conduct such analyses and contribute to the field.

## Supplementary Material

Supplement 1

## Data Availability

Code to replicate all results will be made available in a publicly accessible data repository. The Medicaid data can be obtained under a data use agreement from the Centers for Medicare & Medicaid Services.

## Footnotes

1 We acknowledge that not all people delivering a child are mothers. Throughout, we analyze all Medicaid beneficiaries who receive care for delivery.

2 For example, we include CHIP and Emergency Medicaid beneficiaries.

3 For example, only beneficiaries who are enrolled for a sufficient period can be included in studies focused on healthcare utilization before and after delivery.

4 We use the term claims to refer to all claims and encounter records, specifically rows from all inpatient and other services files.

5
https://resdac.org/cms-data/variables/delivery-code-max-ip

6 We exclude any case ID numbers assigned to more than 10 beneficiaries in a state-year, as these are unlikely to represent a household (16).

7 Past work using diagnosis codes to identify care for newborns has identified only slightly more newborns than expected, which is also what we see when only using claims to identify newborns (4). This arises from significant data loss in states that bill newborn care under mother’s insurance with over-coverage in other states. We prefer to identify newborns in all states even if it means that the nationwide total is higher than expected.

8 Note that since the number of newborns exceeds the number of deliveries, the maximum match rate for all newborns would be 90.89%.
