# Supplementary material for: Linking Newborns and Mothers to Enable the Study of Inter-generational Health Outcomes: Evidence from Nationwide Medicaid Data
Source: Res Sq. 2024 Nov 14:rs.3.rs-5327524. Preprint. [Version 1] doi: 10.21203/rs.3.rs-5327524/v1 (PMC11601815; doi:10.21203/rs.3.rs-5327524/v1)
Supplement: Supplement 1 [file NIHPPRS5327524V1-supplement-1.pdf]

Supplementary Files

This is a list of supplementary files associated with this preprint. Click to download.

- Appendix.pdf
